# Use of Food Delivery Applications and Their Perceived Effects Among Students at a Postgraduate Public Health Institute in Kolkata: A Mixed-Methods Study

**DOI:** 10.7759/cureus.97323

**Published:** 2025-11-20

**Authors:** Kirtesh Tiwari, Jayita Pal, Sanjoy Sadhukhan, Koustab Ghosh, Rajdeep Shaw, Lamabam H Singh

**Affiliations:** 1 Epidemiology and Public Health, All India Institute of Hygiene and Public Health, Kolkata, IND

**Keywords:** college students, eastern india, emotional eating, mixed methods, obesity, online food delivery

## Abstract

Background: Food delivery applications (FDAs) have transformed dietary behavior by providing convenience and diverse food options, particularly among students. As future public health professionals, the food habits of public health students hold implications for population health promotion and policymaking. This study aimed to assess the usage patterns and perceived effects of FDAs among students of the All-India Institute of Hygiene and Public Health and to examine their association with nutritional status (BMI). It also explored reasons underlying high app usage, expenditure, and BMI.

Materials and methods: An institution-based, mixed-method, explanatory sequential study was conducted from March to July 2025 among 106 students selected by census sampling. Quantitative data were collected using a pretested, semi-structured questionnaire and anthropometric measurements, followed by qualitative in-depth interviews (n = 10) with purposively selected participants who exhibited high app usage, expenditure, or BMI.

Results: Among 106 students, 84% reported using FDAs, with monthly orders (50.6%) and a median expenditure of ₹2000 (IQR 2327). Most users were overweight or obese (77%), including 35.8% overweight, 34.9% obese I, and 6.6% obese II. Students with higher app usage frequency (≥10 orders/3 months) had a higher obesity prevalence (46%) than those with lower frequency (31%). The most commonly perceived health problems were weight gain (23.6%), indigestion (23.6%), and irregular meal timing (22.4%). While 92% valued FDAs for saving time, 74% acknowledged their role in overeating, 81% linked them to sedentary habits, and 63% felt they encouraged solitary eating. Qualitative insights revealed that convenience, stress-related comfort eating (48%), and social isolation (60%) reinforced frequent use, forming a cycle connecting emotional coping, unhealthy dietary habits, and rising BMI.

Conclusions: Frequent use of FDA products among students is associated with unhealthy dietary choices, weight gain, and social isolation. Targeted health promotion strategies are warranted to foster mindful, balanced app use among future public health professionals.

## Introduction

Food delivery applications (FDAs) are among the fastest-growing segments of the food industry in India. These platforms may be restaurant-controlled, independent, or operated as large-scale online food delivery (OFD) services such as Swiggy and Zomato. Compared with home-prepared meals, restaurant foods are typically higher in calories, saturated fat, and sodium, while being less nutritionally dense [1]. Despite the convenience they offer, the increasing use of FDAs has raised significant public health concerns. Previous studies have described these platforms as “junk food on demand,” highlighting their role in facilitating easy access to fast food and calorie-dense restaurant meals [2,3]. OFD has also been linked to unhealthy dietary behaviors and reduced physical activity [4].

In recent years, students have become frequent users of FDAs, often purchasing high-calorie and nutrient-poor meals that may adversely affect their health and well-being [5]. Globally, the adoption of OFD services has surged, particularly during the COVID-19 pandemic, when restaurant food delivery grew by 47% in 2020 and over 1.6 billion people used OFD services in 2021 [6]. In Asia, countries such as China experienced a dramatic increase in OFD adoption, from 16.5% in 2015 to 52.7% in 2021, while Malaysia has seen steady expansion driven by new service providers [7].

Students are especially susceptible to unhealthy dietary habits such as irregular meal times, uncontrolled portion sizes, and frequent consumption of high-calorie foods. The persistence of these behaviors may increase vulnerability to non-communicable diseases (NCDs) at a younger age [8,9]. Despite the widespread use of FDAs, limited research examines their effects among students, particularly on body mass index (BMI) and perceived health impacts [10]. The rapid growth of these platforms has been driven by smartphone penetration, busy academic schedules, and limited availability of healthy food options on campuses, making FDAs a convenient choice for students [11].

The proliferation of FDAs has transformed meal consumption patterns, offering convenience while posing potential risks to diet quality and weight management. Understanding the patterns of FDA use and their perceived effects among postgraduate public health students is particularly relevant, as these individuals represent future public health professionals. Increasing awareness of the potential health risks associated with frequent FDA use among this group could yield broader public health benefits. In the Indian context, particularly in Kolkata, there is a paucity of research exploring this topic. This study aims to fill this gap by examining the use and perceived effects of FDAs among students at a postgraduate public health institute in Kolkata, thereby contributing to an understanding of how digital food environments may influence lifestyle and NCD risk among young adults.

## Materials and methods

This institution-based observational study employed a mixed-methods explanatory sequential design, comprising an initial quantitative cross-sectional phase followed by a qualitative phase of in-depth interviews. The study was conducted at the All India Institute of Hygiene and Public Health in Kolkata between March and September 2025.

The quantitative phase included all postgraduate students enrolled in MD (Community Medicine), MD (MPH Epidemiology), M.Sc. (Applied Nutrition), M.Sc. (Public Health), MVPH, and Diploma courses during the 2024-2025 academic year. Students who provided written informed consent were included, while those who declined participation were excluded. A census approach was adopted, and 106 of 118 eligible students completed the survey after accounting for incomplete responses.

Data were collected using a pretested, semi-structured, self-administered questionnaire that captured sociodemographic characteristics, patterns of FDA use, and perceived health and social effects. The instrument was developed in English based on a review of existing literature and previously validated tools. Content and face validity were ensured through expert evaluation by faculty members in Community Medicine and Public Health and through a pilot test among 10 students outside the study sample, which assessed comprehension, feasibility, and completion time. The questionnaire's reliability was established with a Cronbach’s alpha coefficient of 0.82, indicating good internal consistency.

Anthropometric measurements were obtained to calculate BMI. Weight was measured using a calibrated analogue weighing scale (±0.5 kg), and height was measured with a measuring tape to the nearest 0.1 cm. BMI was computed as weight in kilograms divided by height in meters squared and classified according to the World Health Organization Asian cut-offs: underweight (<18.5 kg/m²), normal (18.5-22.9 kg/m²), overweight (23.0-24.9 kg/m²), obese I (25.0-29.9 kg/m²), and obese II (≥30 kg/m²).

Given the census design, all eligible students were included in the study. Descriptive statistics were primarily used, with continuous variables summarized as means and standard deviations and categorical variables as frequencies and percentages. Although inferential testing is generally not required in a census, p-values were calculated to explore internal associations within the study cohort. Chi-square tests were applied for variables with multiple categories, and Fisher’s exact tests were used for 2×2 tables or when expected cell counts were small. These inferential statistics are presented solely for exploratory interpretation within the study population and are not intended for generalization beyond this cohort. For the quantitative analysis, data were analyzed using Excel 2016 (Microsoft Corp., Redmond, WA, USA) and Jamovi version 2.3.28 (The Jamovi Project Pty Ltd., Sydney, Australia), an open-source, user-friendly statistical software. It was used to compute descriptive statistics, correlations, and inferential tests relevant to the study objectives.

Following the quantitative analysis, a qualitative phase was undertaken to explore participants' underlying motivations and perceptions regarding FDA use among those at higher risk. Ten participants were purposively selected based on BMI ≥23 kg/m², total FDA expenditure exceeding ₹5000 during the preceding three months, and more than 10 orders placed within the same period.

An in-depth, semi-structured interview guide was developed from the quantitative findings to explore motivations for FDA use, perceptions of food healthiness, emotional triggers, social influences, and lifestyle modifications. The guide underwent expert review for content and face validity and was piloted with one student outside the study sample. Interviews were conducted in private settings, lasted 25-40 minutes, and were audio-recorded with informed consent. All interviews were transcribed verbatim, and data collection continued until thematic saturation was achieved.

Qualitative data were analyzed manually using thematic analysis following Braun and Clarke’s six-step framework [12]. The process involved familiarization with the transcripts, generation of initial codes, identification and review of emerging themes, and refinement into final thematic categories. Manual analysis was chosen to allow the researcher to engage deeply with the data, ensuring a nuanced understanding of participants’ experiences and contextual factors related to high FDA usage. A second researcher independently reviewed coding and emerging themes, and triangulation with quantitative results was used to enhance the overall validity and interpretation of the findings.

The study was approved by the Institutional Ethics Committee of the All India Institute of Hygiene and Public Health (approval number: IEC/2025(1)/151). Participation was voluntary, written informed consent was obtained from all participants, and confidentiality and anonymity were maintained throughout the study. Participants were informed that they could withdraw at any stage without penalty.

## Results

A total of 106 postgraduate students participated in the study, yielding a response rate of 89.8%. Most participants were aged 26-30 years (40.5%), followed by those aged 31-35 years (25.4%), with a slight male predominance (55.7%). The majority were from urban areas of West Bengal (67.9%) and resided with their families (61.3%). Nearly half of the students (49.1%) were pursuing an MD in Community Medicine, and two-thirds (67.9%) reported receiving stipends. Among the 89 respondents who reported using FDAs, half (50.6%) ordered food once per month, with Zomato (50.6%) and Swiggy (49.4%) being equally preferred. The most commonly cited reason for app use was comfort eating when feeling low or sad (48.3%), followed by attraction to discounts and promotional offers (29.2%). Snacks (39.3%) were the most frequently ordered food category, and Indian cuisine (49.4%) was the most preferred. Most users (71.9%) reported very low usage, defined as five or fewer orders in three months, and over half (50.5%) spent ₹2000 or less during this period (Table 1).

**Table 1:** Sociodemographic, academic, and FDA usage profile of the students (N = 106) Continuous variables are summarized as mean ± SD, median (IQR), or range, as appropriate. Percentages are based on valid responses (N = 106 for the total sample; n = 89 for app users). SD: standard deviation, IQR: interquartile range, FDA: food delivery application

Variable	Category	n (%)	Descriptive statistics
Age (years)	21–25	26 (24.5)	Range: 22–48 years
	26–30	43 (40.5)	Mean ± SD: 29.4 ± 5.08 years
	31–35	27 (25.4)	
	>35	10 (9.6)	
Gender	Male	59 (55.7)	
	Female	47 (44.3)	
Residence	Urban, West Bengal	72 (67.9)	
	Urban, outside West Bengal	20 (18.9)	
	Rural, West Bengal	13 (12.3)	
	Rural, outside West Bengal	1 (0.9)	
Living arrangement	With family	65 (61.3)	
	Hostel	41 (38.7)	
Stream	MD (community medicine)	52 (49.1)	
	MD (MPH – epidemiology)	20 (18.9)	
	M.Sc. (applied nutrition)	16 (15.1)	
	MVPH	14 (13.2)	
	SHI	4 (3.8)	
Stipend	Yes	72 (67.9)	
	No	34 (32.1)	
Frequency of FDA usage (n = 89)	Monthly	45 (50.6)	
	Weekly	32 (36.0)	
	Fortnightly	4 (4.5)	
	Once in 3 months	4 (4.4)	
	2–3 times/week	3 (3.4)	
	Daily	1 (1.1)	
Preferred FDA (n = 89)	Zomato	45 (50.6)	
	Swiggy	44 (49.4)	
Common reasons for app usage (n = 89)	Comfort choice when feeling low/sad	43 (48.3)	
	Discounts and offers	26 (29.2)	
	Unavailability of preferred food	9 (10.2)	
	Social reasons	6 (6.7)	
	Avoiding restaurant queues	5 (5.6)	
Usual meal ordered on FDAs (n = 89)	Snacks	35 (39.3)	
	Lunch	21 (23.6)	
	Breakfast	15 (16.9)	
	Dinner	15 (16.9)	
	Dessert	2 (2.2)	
	Beverages	1 (1.1)	
Preferred cuisine (n = 89)	Indian	44 (49.4)	
	Chinese	22 (24.7)	
	Culinary exploration	17 (19.2)	
	Italian	6 (6.7)	
Usage of FDAs (orders in the last 3 months) (n = 89)	Very low (≤5)	64 (71.9)	
	Low (≤10)	15 (16.9)	
	High (≤20)	7 (7.9)	
	Very high (>20)	3 (3.3)	
Expenditure (₹, last 3 months) (n = 89)	Low (≤2000)	45 (50.5)	Range: 300–24000; median (IQR): 2000 (2327)
	Average (≤5000)	28 (31.5)	
	High (>5000)	16 (18.0)	

Among the students, 21.7% had normal BMI, while the majority (77%) were overweight or obese: 35.8% overweight, 34.9% obese I, and 6.6% obese II. Students with higher app usage frequency (≥10 orders in 3 months) had a higher obesity prevalence (46%) than those with lower frequency (31%), suggesting a possible link between frequent app-based ordering and higher BMI. However, no statistically significant associations were found between BMI category and any sociodemographic or app-related factors (p > 0.05). Although overweight and obesity were more common among older students and those with higher usage and expenditure, these variations were not significant. The absence of significant associations may be attributed to the small cohort size and the census design, in which inferential testing was exploratory rather than intended for population-level generalization (Table 2).

**Table 2:** Distribution of students according to BMI, sociodemographic, and app-related factors (N = 106) p-values marked with * were obtained using the Chi-square test. p-values marked with # were obtained using Fisher’s exact test. Significance level set at p < 0.05. BMI: body mass index, FDA: food delivery application, χ²: Chi-square test, OR: odds ratio, df: degrees of freedom

Factor	Category	N	Normal + underweight n (%)	Overweight + obese n (%)	Test/statistic	p-value
Age range (years)	21–25	26	8 (30.8)	18 (69.2)	χ² = 2.164 (df = 3)	0.539*
	26–30	43	10 (23.3)	33 (76.7)		
	31–35	27	5 (18.5)	22 (81.5)		
	>35	10	1 (10.0)	9 (90.0)		
Gender	Male	59	14 (23.7)	45 (76.3)	OR = 1.15 (0.45–2.89)	0.819#
	Female	47	10 (21.2)	37 (78.8)		
Using FDA	Yes	89	18 (20.2)	71 (79.8)	OR = 2.15 (0.70–6.60)	0.208#
	No	16	6 (35.3)	10 (64.7)		
Living arrangement	Hostel	39	9 (23.1)	30 (76.9)	OR = 1.04 (0.41–2.63)	1.00#
	With family	67	15 (22.4)	52 (77.6)		
Frequency of FDA usage	Monthly	45	10 (22.2)	35 (77.8)	χ² = 3.606 (df = 7)	0.824*
	Weekly	32	8 (25.0)	24 (75.0)		
	Fortnightly	4	0 (0.0)	4 (100)		
	Once in 3 months	4	0 (0.0)	4 (100)		
	2–3 times/week	3	0 (0.0)	3 (100)		
	Daily	1	0 (0.0)	1 (100)		
Starting age of FDA use (years)	<20	11	3 (27.3)	8 (72.8)	χ² = 2.896 (df = 3)	0.408*
	21–25	38	7 (18.5)	31 (81.5)		
	26–30	32	8 (25.0)	24 (75.0)		
	>30	8	0 (0.0)	8 (100)		
Receiving stipend	Yes	72	15 (20.8)	57 (79.2)	OR = 0.73 (0.28–1.91)	0.620#
	No	34	9 (26.4)	25 (73.6)		
Expenditure pattern (₹, last 3 months)	Low (≤2000)	45	11 (24.4)	34 (75.6)	χ² = 2.458 (df = 2)	0.293*
	Average (≤5000)	28	6 (21.4)	22 (78.6)		
	High (>5000)	16	1 (6.3)	15 (93.7)		

Perceptions of FDA use revealed mixed awareness of its health and lifestyle implications. The most commonly perceived health problems associated with app use included increased body weight (23.6%), indigestion (23.6%), and irregular meal timing (22.4%), followed by heartburn (12.4%). A smaller proportion reported psychosomatic concerns such as loneliness (5.6%), insomnia (4.5%), appetite changes (4.5%), and altered bowel habits (3.4%). Nearly half (46%) of users felt that healthy food options were rarely available on these platforms, and 83% believed that their typical orders were unhealthy. Additionally, 74% of respondents agreed that FDAs promote overeating to some extent or more, and 59% felt that app use reduced social interactions. Nevertheless, 92% viewed FDAs as time-saving and convenient, especially amid demanding academic schedules. A majority (81%) acknowledged that regular app use contributes to a sedentary lifestyle, and 63% believed it encourages solitary eating. Notably, 96% of participants agreed that reducing app usage could save money. These findings indicate that while FDAs provide convenience, they also contribute to unhealthy dietary behaviors and social isolation among students (Table 3).

**Table 3:** Perception of FDA usage among students (n = 89) FDA: food delivery application

Statement	Cannot say n (%)	Absolutely not n (%)	To some extent n (%)	Most likely n (%)	Yes, definitely n (%)
Students felt that healthy food options were available on FDAs	6 (6.7)	41 (46.1)	41 (46.1)	1 (1.1)	0 (0.0)
Students believed that their food orders through apps were healthy	1 (1.1)	74 (83.1)	13 (14.6)	1 (1.1)	0 (0.0)
Students perceived that FDAs contribute to overeating	4 (4.5)	19 (21.3)	38 (42.7)	25 (28.1)	3 (3.4)
Students felt that FDAs affect their social interactions	10 (11.3)	26 (29.2)	34 (38.2)	18 (20.2)	1 (1.1)
Students agreed that FDAs help save time for other activities	2 (2.3)	5 (5.6)	41 (46.1)	31 (34.8)	10 (11.2)
Students believed that FDAs contribute to a sedentary lifestyle	2 (2.2)	14 (15.7)	34 (38.2)	32 (36.0)	7 (7.9)
Students agreed that FDAs encourage solitary eating habits	3 (3.4)	30 (33.7)	30 (33.7)	22 (24.7)	4 (4.5)
Students perceived that reducing the use of FDAs could save money	0 (0.0)	3 (3.4)	29 (32.5)	47 (52.8)	10 (11.3)

To explore these patterns in greater depth, qualitative interviews were conducted with 10 purposively selected participants who met the following criteria: BMI ≥23 kg/m², expenditure exceeding ₹5000 on FDA orders in the past three months, and more than 10 orders placed during the same period. The sample included six males and four females aged 27-33 years, with BMI ranging from 24.5 to 30.6 kg/m². Most reported weekly or several-times-weekly app use, with a few ordering daily (Table 4).

**Table 4:** Sociodemographic and FDA usage pattern of students of qualitative strand (n = 10) BMI: body mass index, FDA: food delivery application, M: male, F: female

Participant ID	Age (years)	Gender	No. of orders (last 3 months)	Expenditure (₹)	BMI (kg/m²)	FDA usage frequency
1	29	M	60	13,200	26.4	Daily
2	33	M	11	6,000	29.4	Weekly
3	28	M	14	5,400	27.7	Fortnightly
4	28	M	20	10,000	24.5	2–3 times a week
5	32	M	37	18,000	30.6	2–3 times a week
6	27	M	30	24,000	26.8	Weekly
7	30	F	14	6,300	27.9	Weekly
8	29	F	18	5,700	29.5	Weekly
9	28	F	15	5,500	25	Weekly
10	30	F	16	8,000	29	Weekly

Thematic analysis of interview transcripts identified four major themes explaining high FDA usage, elevated BMI, and increased expenditure: (1) convenience and accessibility, (2) unhealthy dietary patterns, (3) social isolation (loneliness), and (4) comfort choice during stress or sadness (Table 5).

**Table 5:** Thematic analysis of the qualitative strand (N = 10) Themes were derived using Braun and Clarke’s six-step framework for thematic analysis. Verbatim quotes are included to illustrate each theme. FDA: food delivery application

Theme	Description	Representative quotable quotes
1. Convenience and accessibility	Participants viewed FDAs as a time-saving and effortless option that fit well with their demanding academic schedules. The ease of ordering and access to multiple cuisines made FDAs preferable to cooking or dining in canteens.	“I can get food in 20 minutes without stepping out — that’s a big relief when I’m tired.”
2. Unhealthy dietary patterns	Frequent FDA use led to higher intake of calorie-dense and nutrient-poor foods such as pizza, fried snacks, and desserts. Discounts and offers encouraged impulsive buying and overeating, contributing to gradual weight gain.	“Most of the time I end up ordering pizza or fried food, even if I planned to eat healthy. Discounts tempt me to order more, and I usually overeat when the food arrives.”
3. Social isolation (loneliness)	Participants reported that FDA use promoted solitary eating habits and reduced social interactions. Feelings of loneliness or anxiety in communal eating settings led some to prefer eating alone in their rooms.	“Lack of enough friends makes me anxious and feel isolated around a group of friends eating together in the canteen. So, I avoid going to the canteen and order food in my room only.”
4. Comfort choice during stress or sadness	Emotional eating was frequently used as a coping mechanism during stress, sadness, or academic workload. Ordering food was described as comforting and emotionally rewarding.	“Whenever I’m stressed with exams, I crave fast food, ordering feels like a reward. On bad days, I just order something comforting; it instantly lifts my mood.”

Triangulation of quantitative and qualitative data highlighted four principal drivers of frequent FDA use and elevated BMI: convenience and accessibility, unhealthy dietary choices, social isolation, and emotional coping. Among participants with high BMI and frequent app use, 92% perceived FDAs as time-saving, 83% reported ordering unhealthy foods, 80% were overweight or obese, and 60% experienced reduced social interactions. Nearly half (48%) identified comfort eating as a habitual response to stress. Collectively, these findings suggest that convenience, emotional regulation, unhealthy food preferences, and social withdrawal interact to sustain high app usage and contribute to elevated BMI.

**Figure 1:**
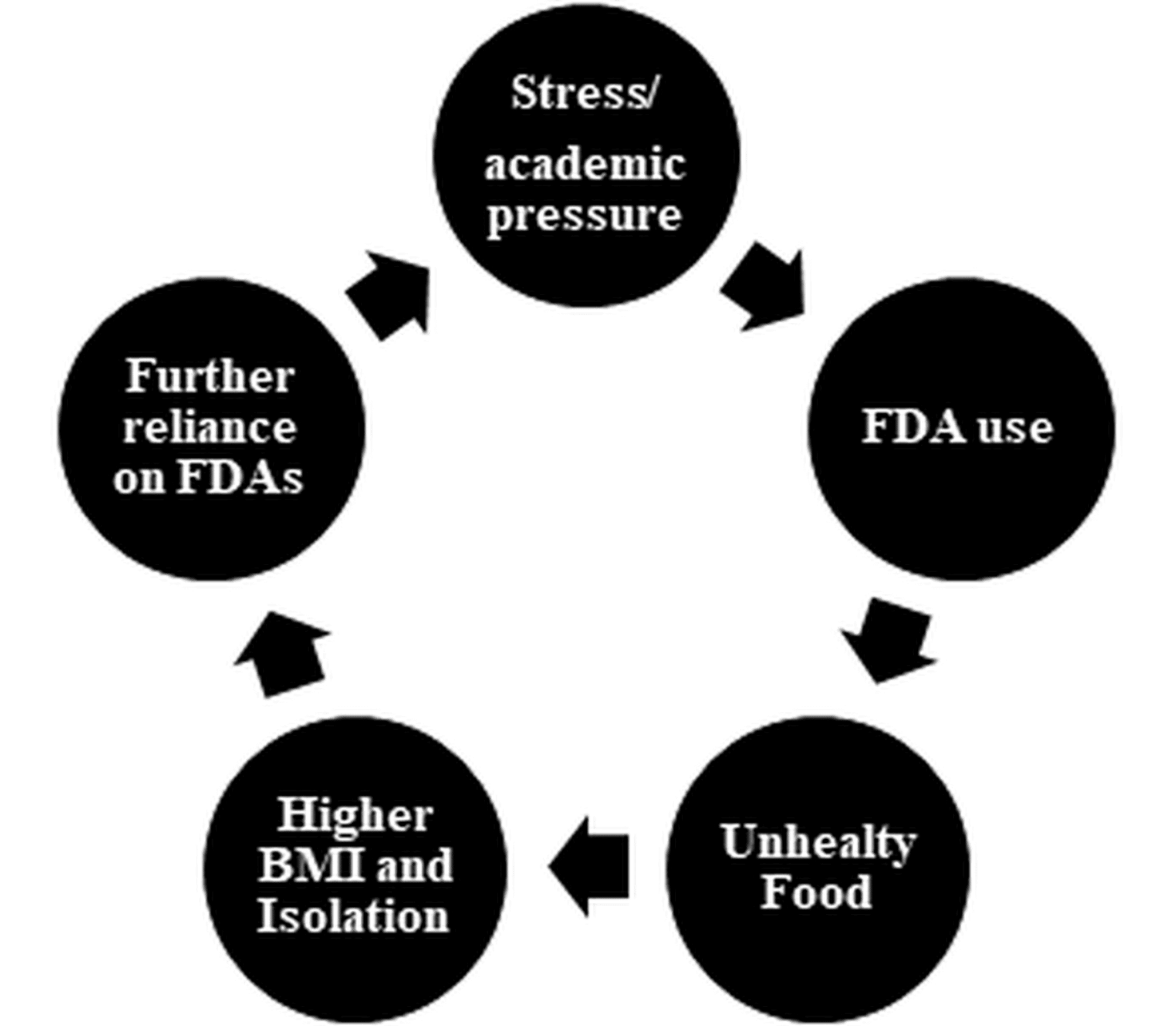
Cycle of FDA use and BMI among students FDA: food delivery application, BMI: body mass index

An integrated interpretation of the findings indicates a reinforcing cycle linking academic stress, frequent use of FDA, and adverse health outcomes. Academic demands and time constraints lead students to rely on FDAs for convenience, promoting the consumption of calorie-dense foods. This behavior contributes to weight gain and reduced social engagement, which in turn fosters emotional eating and further dependence on FDAs. The cycle perpetuates itself as ongoing academic pressure continues to drive app-based ordering and unhealthy dietary habits (Figure 1).

## Discussion

This study explored the use of FDAs and their associations with overweight and obesity, dietary behaviors, and social patterns among postgraduate public health students in Kolkata. A high prevalence of FDA use (84%) and overweight/obesity (77%) was observed. Integration of quantitative and qualitative findings revealed that convenience, stress relief, and promotional incentives were the principal drivers of FDA use, while emotional eating and social isolation further reinforced unhealthy dietary practices.

Convenience and time-saving were the most prominent motivators for app use, as students viewed FDAs as an efficient solution to their academic workload and time constraints. This finding mirrors results from previous studies in India and Malaysia, which identified convenience and busy academic schedules as key determinants of frequent online food ordering [13,14]. The similarity of these patterns across diverse settings suggests that convenience-driven food app use transcends cultural and educational contexts and has become an integral part of student food environments.

Frequent FDA use was strongly associated with unhealthy dietary behaviors and elevated BMI. In this study, approximately 83% of participants reported ordering calorie-dense and nutritionally poor foods, while the majority were classified as overweight or obese. These findings are consistent with the observations of Srivastava et al. (2021), who reported increased BMI among medical and dental students using FDAs, and Dai et al. (2022), who found that meals ordered online in China were typically high in fat and sodium and associated with higher body weight [14,15]. Ab Hamid et al. (2024) similarly documented that students with greater ordering frequency had higher BMI despite awareness of unhealthy food choices [10]. Collectively, this evidence supports a global trend in which frequent FDA use contributes to overweight and obesity through increased energy intake, disrupted meal regularity, and persistent preference for calorie-dense foods, even among health-literate populations.

The qualitative findings revealed that FDA use also served as an emotional coping mechanism. Nearly half of the participants reported ordering food when feeling stressed or sad, supporting the “treat-yourself” model proposed by Capito and Pergelova (2023) [16]. Comparable results were reported by Buettner et al. (2023) and Willie et al. (2024), who found that food apps acted as emotional regulators and reinforced sedentary behavior and unhealthy eating patterns [17,11]. The present study extends this evidence by directly linking stress-related food app use to elevated BMI, demonstrating that emotional and behavioral drivers interact to influence physical health outcomes.

Social isolation also emerged as an important behavioral correlate. Many students preferred solitary eating facilitated by FDAs and avoided shared dining spaces, resulting in fewer social interactions. This pattern is consistent with findings from Vaidya et al. (2025), who described individualization of food consumption among students, and Selvan et al. (2021), who highlighted the rise of a digital “eating-in” culture [13,18]. Such solitary eating behaviors may further perpetuate dependence on FDAs, reinforcing both emotional eating and reduced physical activity.

Triangulation of findings supports a behavioral feedback loop in which academic pressure drives reliance on FDAs, leading to increased consumption of unhealthy foods, higher BMI, and reduced socialization. These outcomes, in turn, heighten stress and further dependence on FDAs. Similar cyclical dynamics have been documented internationally, suggesting that convenience, digital marketing, and emotional coping within digital food environments collectively override nutritional awareness and promote unhealthy eating behaviors [19].

The implications of these findings are significant. FDAs have become an integral component of the modern food environment for young adults, driven by convenience, affordability, and targeted marketing. Among public health students, who are expected to model healthy lifestyles, the high prevalence of unhealthy ordering highlights the need for comprehensive interventions. Strategies could include integrating digital nutrition education into curricula, promoting stress management and mindful eating, facilitating structured meal planning, and encouraging communal dining. Addressing behavioral and environmental determinants is essential to mitigate long-term health risks associated with frequent FDA use, even among populations with strong health literacy.

This study’s primary strength lies in its mixed-methods design, which combines quantitative data with qualitative insights to provide both breadth and depth of understanding. Examining FDA usage among a health-literate cohort offers a unique perspective on behavioral gaps between knowledge and practice. The inclusion of national and international comparative evidence enhances the contextual relevance of the findings, while the post-pandemic timing captures current food environment trends that are highly policy-relevant.

However, certain limitations must be acknowledged. Data were self-reported, which may introduce recall or social desirability bias regarding ordering frequency and dietary perceptions. The study was conducted at a single institution, limiting geographic generalizability, and the census-based qualitative strand restricts the use of statistical significance testing. Since the study employed a census design, formal statistical inference is limited. The reported p-values should therefore be interpreted as descriptive indicators of within-cohort associations rather than evidence of generalizable relationships. Additionally, dietary quality was assessed based on self-perception rather than objective nutrient or caloric analysis, which may affect the precision of dietary evaluations.

## Conclusions

This study underscores the complex interplay of behavioral, emotional, and digital factors influencing FDA use among public health students. Convenience, emotional coping, and digital marketing were found to collectively shape dietary choices, contribute to higher BMI, and promote social isolation. Triangulation of quantitative and qualitative findings revealed a reinforcing cycle in which academic pressure, app dependency, and unhealthy eating behaviors perpetuate one another, consistent with evidence from both Indian and international research. These results demonstrate that even health-literate populations remain vulnerable to technology-driven dietary risks.

To mitigate these challenges, academic institutions should incorporate digital nutrition literacy and stress-management training into curricula, organize workshops on mindful eating, and provide targeted counseling for emotional eating. Public health interventions should also leverage social media to promote balanced diets, portion control, and digital self-awareness. Future studies are recommended to adopt longitudinal and multi-center designs with objective dietary tracking and to explore socio-demographic variations in FDA-related behaviors. Strengthening these approaches will be essential to reducing the long-term health and social consequences of excessive use of FDAs among young adults.
